# Effect of Foot Reflexology on Restless Legs Syndrome and Sleep Quality in Female Patients With Multiple Sclerosis: A Randomized, Double‐Blind Clinical Trial

**DOI:** 10.1155/nri/5579094

**Published:** 2026-07-15

**Authors:** Fatemeh Harandi, Batool Tirgari, Yunes Jahani, Haleh Tajadini

**Affiliations:** ^1^ Physiology Research Center, Institute of Neuropharmacology, Kerman University of Medical Sciences, Kerman, Iran, kmu.ac.ir; ^2^ Nursing Research Center, Kerman University of Medical Sciences, Kerman, Iran, kmu.ac.ir; ^3^ Modelling in Health Research Center, Institute for Futures Studies in Health, Kerman University of Medical Sciences, Kerman, Iran, kmu.ac.ir; ^4^ Neuroscience Research Center, Institute of Neuropharmacology, Kerman University of Medical Sciences, Kerman, Iran, kmu.ac.ir

**Keywords:** multiple sclerosis, reflexology, restless leg syndrome, sleep quality

## Abstract

**Background:**

This clinical trial aimed to assess the effectiveness of foot reflexology on restless legs syndrome and sleep quality in patients with multiple sclerosis.

**Materials and Methods:**

Sixty‐four female patients with multiple sclerosis were randomly assigned to the intervention or sham group. Foot reflexology was taught to the intervention group; it was performed for 4 weeks, three times a week for 26 min each session, by a trained researcher in the morning shift and on three other days in the afternoon at home by the patients themselves. Moreover, the sham group received nonspecific foot massage without applying pressure to the standard reflexology points under similar conditions and durations as those of the intervention group. Data were collected before, immediately after, and 1 month after intervention.

**Results:**

The results revealed a significant improvement in sleep quality (*p* < 0.001) in the intervention group. No significant change was observed in the sham group (*p* = 0.30). Restless leg syndrome significantly decreased in the intervention group (*p* < 0.001), whereas it increased in the sham group (*p* < 0.001). There was a significant difference in the sleep quality after the intervention between the two groups at both the immediate and 1‐month follow‐ups (*p* < 0.0001). There was also a significant difference in the restless leg syndrome after the intervention between the two groups at both the immediate and 1‐month follow‐ups (*p* < 0.0001).

**Conclusions:**

Nurses should be trained in foot reflexology as a noninvasive approach that can be easily implemented.

**Summary**
• Restless leg syndrome (RLS) is one of the most prevalent conditions among individuals with MS.
• Poor sleep quality is a common problem in MS patients.
• Foot reflexology can effectively improve RLS symptoms and sleep quality in individuals with MS.

## 1. Introduction

Multiple sclerosis (MS) is an autoimmune disease of the central nervous system [1]. Evidence suggests that women are two to three times more likely than men to develop MS [2]. Approximately 2.9 million people worldwide have MS [3]. The prevalence of this disease in Iran is 100 per 100,000 people [4]. The clinical manifestations of MS vary depending on the location and extent of nerve damage, leading to a wide range of motor, sensory, and cognitive impairments that significantly impact quality of life [5–7].

Restless leg syndrome (RLS) is one of the most prevalent conditions among individuals with MS [8]. These patients experience a compelling urge to move their legs, often described as an uncomfortable or even painful sensation. This urge typically worsens during periods of rest, particularly in the evening or night, and often interferes with sleep [9]. While the prevalence of RLS in MS patients has been reported to be approximately 46% in Iran, studies have consistently shown that RLS in MS patients disrupts sleep quality and consequently reduces health‐related quality of life [8, 10, 11]. Poor sleep quality is a common problem in MS patients and is often exacerbated by RLS [12]. Sleep disturbances can negatively impact the overall health and quality of life of these individuals. In fact, it has been reported that up to 75% of MS patients suffer from at least one sleep disorder [13, 14].

Massage therapy is one of the most common and safest complementary therapies worldwide and has been shown to be beneficial for managing conditions such as RLS [15]. Massage can stimulate the cerebral cortex, increase dopamine production, and improve tendon and muscle tension, thereby alleviating RLS symptoms [16, 17]. Reflexology is a type of massage therapy that involves applying pressure to specific reflex points on the body, particularly the feet, to promote relaxation and healing [18, 19]. Reflexology can improve sleep quality by inducing relaxation, reducing anxiety, and alleviating pain [12].

Foot reflexology, as a noninvasive nursing intervention, holds promise as a complementary therapy for managing the symptoms of MS [20]. While some evidence indicates that reflexology can improve sleep quality and reduce symptoms of RLS in the general population [12, 21–23], its specific impact on MS patients has not been extensively studied. Given the high prevalence of RLS and sleep disorders in MS patients, investigating the effectiveness of reflexology in this cohort is particularly pertinent. Prior exploratory studies have suggested that reflexology may help reduce the severity of RLS symptoms and promote better sleep quality by enhancing circulation, reducing muscle tension, and promoting relaxation [12, 17, 22, 24, 25]. However, rigorous clinical trials focusing on MS patients are scarce, highlighting the need for well‐designed studies to validate these preliminary findings. Therefore, this clinical trial aimed to assess the effectiveness of foot reflexology on RLS and sleep quality in patients with MS.

### 1.1. Objective

To evaluate whether foot reflexology improves sleep quality and RLS in patients with MS.

## 2. Materials and Methods

### 2.1. Study Design and Setting

This study is a double‐blind clinical trial. The study population consisted of female patients with MS who were referred to the Medical Center of the Special Diseases in Kerman in 2023. Neither the statistician nor the participants were aware of which data belonged to the intervention or the sham group.

### 2.2. Sample Size and Sampling

The sample size was calculated via the formula for comparing two independent means on the basis of data from Ghanbari et al. [22], with *α* = 0.05, 90% power, standard deviations of 1.07 and 1.95 for the intervention and sham groups, respectively, and a minimum detectable difference of 1.5 points in the RLS score; the calculated sample size was 25 per group. To account for potential dropouts, this percentage was increased by approximately 20%, resulting in a final sample size of 32 patients per group.

**FIGURE 1:**
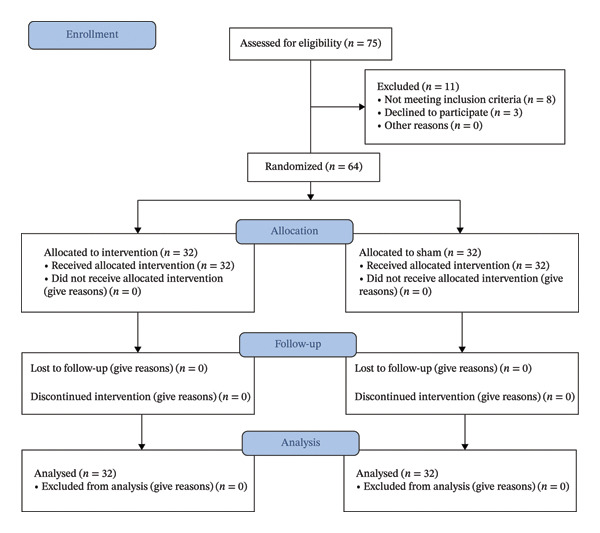
Flowchart of patients.

• A convenience sampling method was employed, and participants were selected on the basis of the inclusion criteria. The participants were randomly assigned to the intervention and sham groups via block randomization. A block size of four was used, with two participants allocated to the intervention group and two to the sham group in each block via R software performed by a statistician. The principal investigator was responsible for both enrollment of participants and the assignment to intervention and sham groups and had access to the random allocation sequence (Figure 1).

The inclusion criteria were as follows: age between 18 and 65 years, female sex, full consciousness and orientation, ability to read, write, and communicate in Persian, diagnosis of MS confirmed based on the patient’s medical records, meeting all four international criteria for diagnosing RLS [22, 26], an RLS severity scale score of 11 or higher, sleep disturbances (total score of 6 or higher on the Pittsburgh Sleep Quality Index [PSQI]), ability to perform daily living activities independently, and intact, undamaged skin on the lower extremities.

The exclusion criteria were as follows: paralysis, sensory disorders, peripheral neuropathy, vascular disorders, coagulation issues, abnormalities, or orthopedic problems in the lower limbs; neurological diseases other than MS; pregnancy; diseases that cause RLS, such as chronic kidney disease; chronic diseases, such as cancer, chronic respiratory failure, heart failure, and rheumatoid arthritis, on the basis of medical records; the use of medications that worsen RLS, such as selective serotonin reuptake inhibitors, antiemetics, anticonvulsants, and dopamine antagonists; previous experience with foot reflexology for treating RLS or sleep disorders; and the use of other complementary and alternative therapies and alcohol or drug addiction.

### 2.3. Instruments

#### 2.3.1. Demographic and Medical Information Questionnaire

A questionnaire was developed to collect demographic and medical information. The demographic data included age, marital status, education level, economic situation, and job. Medical information included the type of disease, medications used, duration of MS, and recurrence in the past year [22, 27].

#### 2.3.2. PSQI

This scale was used to assess sleep quality. The PSQI consists of nine questions related to seven components, including subjective sleep quality, sleep latency, sleep duration, habitual sleep efficiency, sleep disturbances, use of sleep medication, and daytime dysfunction. Each question is scored on a scale of 0 to 3, with a total score ranging from 0 to 21. Higher scores indicate poorer sleep quality. A score of 5 or greater is considered indicative of poor sleep quality [28]. The validity and reliability of this questionnaire have been established in various studies. The Persian version of this questionnaire, validated by Mezerji et al. [29] in Iran, demonstrated an excellent item content validity index (≥ 0.78) and an excellent scale content validity index (≥ 0.90), with a Cronbach’s alpha of 0.65 [29].

#### 2.3.3. International RLS Study Group (IRLSS) Questionnaire

This questionnaire was used to assess the severity of RLS. The first part of the questionnaire includes four questions to confirm the diagnosis of RLS, and all four questions must be answered affirmatively. These questions include the following: (1) During the past month, have you had any uncomfortable sensations or an urge to move your legs at rest? (2) During the past month, did uncomfortable sensations or an urge to move your legs occur or worsen when you were sitting or lying down? (3) During the past month, was the urge to move your legs or uncomfortable sensations relieved by movement, such as walking around? (4) During the past month, did uncomfortable sensations or an urge to move your legs worsen in the evening or at night compared with during the day? The second part assesses the severity of RLS via ten five‐point Likert scale items, with total scores ranging from 0 to 40. Higher scores indicate greater severity of RLS. The severity of this disorder is classified into five categories on the basis of the scores obtained: no problem (0), mild (1–10), moderate (11–20), severe (21–30), and very severe (31–40) [11]. In a study conducted in Iran to determine the validity and reliability of the Persian version of the RLS diagnostic questionnaire, content validity was assessed first, and Cronbach’s alpha coefficient (0.90) was used to determine the reliability of this questionnaire. The reliability of the RLS severity questionnaire was evaluated by calculating Cronbach’s alpha coefficient of 0.97 [30].

### 2.4. Data Collection and Intervention

Patients were selected according to the inclusion criteria. The severity score of RLS was assessed on the basis of the IRLSS questionnaire, and patients were included in the study if their score was 11 or higher. Additionally, sleep quality was measured via the PSQI, and patients with a total score of 6 or higher on the basis of the PSQI were included in the study. The PSQI questionnaire was administered by the researcher, and participants were instructed to respond based on their recent sleep experience during the intervention period. The recruitment period for the study extended from 31 July 2023 to 31 October 2023. All enrolled participants were followed for one month after recruitment to assess study outcomes.

The principal investigator received training in foot reflexology and conducted the intervention 3 days per week during the morning shift. Considering that RLS symptoms often worsen in the afternoon (3:00 p.m.–7:00 p.m.) [31, 32] and due to the inability of patients to visit the center during this time, the researcher provided reflexology training, guiding them to find a comfortable position. They were advised to sit in a kneeling position on the floor or on a chair, placing one arm on the other for easy access to massage their foot. The patients practiced reflexology in the presence of the researcher, and their questions were answered. Additionally, videos of the reflexology procedure were recorded by the patients, and any ambiguities and issues were addressed in the subsequent sessions. They were then asked to perform foot reflexology at home in the afternoon on three other days of the week according to the training they received, except for the 3 days when the researcher conducted the intervention in the morning shift. Since the PSQI assesses sleep quality over the past month, a 4‐week intervention period was considered.

**FIGURE 2:**
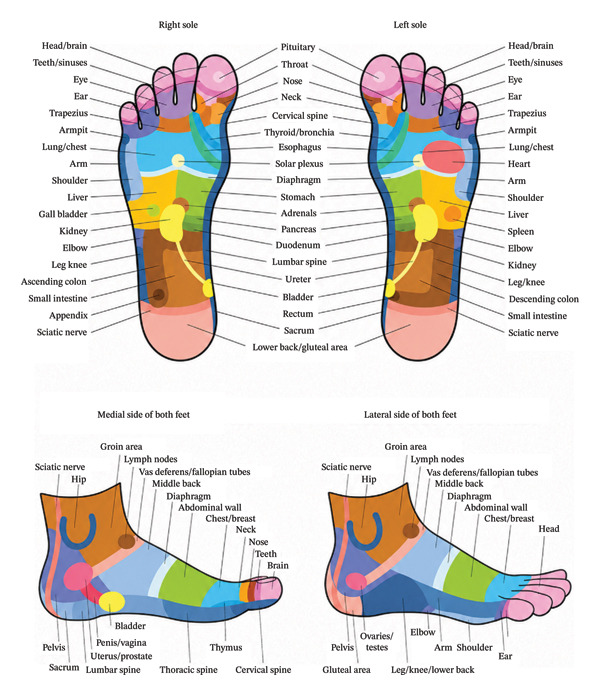
Reflex points on the sole of the foot.

In both groups, before the intervention, all necessary measures, such as maintaining privacy and appropriate verbal communication with the patient, were observed. The patient was positioned face down on the bed in a calm environment with adequate lighting and a suitable temperature. The researcher sat on a chair at the foot of the patient’s bed and performed the massage. To prevent fatigue, a small pillow was placed under the knees. The intervention groups received general foot reflexology, with a focus on 21 reflex points on the sole of the foot [33] (Figure 2). One milliliter of baby oil (Firooz Co., Iran) was used for each leg to facilitate massage. For the first 3 minutes of each session, a soothing massage was performed by both the researcher and the patient at home to prepare for the main technique. For the main technique, the heel of one foot was grasped with the left hand, and the tip of the right thumb was used to apply intermittent wave‐like pressure to the corresponding reflex points on the sole of the foot. The massage was performed slowly, rhythmically, and at a depth that was comfortable for the patient. Since the movements were performed in a wave‐like manner from one point to another, 1 minute of the protocol was dedicated to these rotational movements. Finally, 2 minutes of simple massage were performed for relaxation by both the researcher and the patient at home.

This protocol was carried out for 26 min per session (13 min per foot) [12, 17, 22, 31, 34], three times a week for four weeks, and was repeated three additional times by the patient at home [33, 35].

For the sham group, simple foot massage without pressure on the standard reflexology points was performed 3 days per week for 4 weeks under the same conditions and duration as the intervention group. Patients in the sham group were also instructed to perform the massage at home in the afternoon on three other days of the week. They were asked to perform the massage at home in the afternoon on three other days of the week, except for the 3 days when the researcher performed the massage during the morning shift (for a duration of 4 weeks). To prevent interference between the intervention and sham groups, foot reflexology was performed on even‐numbered days for the intervention group, whereas massage was performed on odd‐numbered days for the sham group by the researcher.

### 2.5. Harm Monitoring

Potential harms were defined prior to the study and assessed systematically using participant self‐reports and continuous monitoring during all intervention sessions.

### 2.6. Data Analysis

The data were entered into SPSS Version 26. Descriptive statistics, including means, standard deviations, frequencies, and percentages, were used to describe the data. For comparing demographic variables, Fisher’s exact test, the chi‐square test, and the independent samples *t* test were employed. Normality of the numerical variables was evaluated using skewness, kurtosis, Q–Q plots, and the Shapiro–Wilk test, all of which indicated that the normality assumption was satisfied. To compare changes in sleep quality and RLS scores before and after the intervention within each group, repeated measures analysis of variance (RM ANOVA) was used. To perform pairwise comparisons between time points in each group, the Bonferroni post hoc correction was used. To compare the RLS and sleep quality scores between the intervention and sham groups at the immediate and 1‐month follow‐ups, RM ANOVA was also used. The level of significance was set at 0.05.

### 2.7. Sensitivity Procedure

Given the marked improvement in RLS severity scores and to ensure a robust interpretation of treatment response, a sensitivity analysis was conducted. Participants with complete symptom resolution (postintervention score = 0) were excluded, and the analysis was repeated in the remaining cohort.

### 2.8. Missing Data

No missing data were observed for the outcomes analyzed in this study.

## 3. Data Quality Assurance

To ensure the accuracy and reliability of the data, several quality control procedures were implemented throughout the study. All data were entered independently and then cross‐checked against the original data collection forms to minimize entry errors. The recorded values were reviewed for completeness and consistency, and any discrepancies were resolved by referring back to the source documents. Standardized procedures were followed for scoring all outcome measures, including the IRLS and PSQI questionnaires, to ensure uniformity across participants. These steps were taken to enhance the overall validity and integrity of the dataset.

## 4. Results

### 4.1. Descriptive Results

This study analyzed data from 64 female patients diagnosed with MS. The intervention and sham groups were matched in terms of potential confounding variables. Table 1 shows no statistically significant differences between the two groups (*p* > 0.05), indicating that they were homogeneous prior to the intervention.

**TABLE 1:** Comparison of demographic and clinical variables between groups.

Variable		Intervention *N* (%)	Sham *N* (%)	Statistic	*p*
Marital status	Single	4 (12.5)	6 (18.7)		0.85
	Married	23 (71.9)	22 (68.8)		
	Other	5 (15.6)	4 (12.5)		

Education level	High school	6 (18.8)	4 (12.5)	0.73^∗^	0.69
	Diploma	14 (43.7)	17 (53.1)		
	Above diploma	12 (37.5)	11 (34.4)		

Economic situation	Good	5 (15.6)	3 (9.4)		0.76
	Moderate	19 (59.4)	22 (68.8)		
	Weak	8 (25)	7 (21.8)		

Job	Employed	9 (28.1)	10 (31.3)		0.86
	Housewife	21 (65.6)	19 (59.4)		
	Unemployed	2 (6.3)	3 (9.3)		

Duration of MS	One to 5 years	5 (15.6)	7 (21.9)	0.41^∗^	0.81
	Six to 10 years	12 (37.5)	11 (34.4)		
	11 years and older	15 (46.9)	14 (43.7)		

Recurrence in the past year	None	21 (65.6)	19 (59.4)		0.79
	Once or twice	11 (34.4)	12 (37.5)		
	More than twice	0 (0)	1 (3.1)		

Type of MS	Primary‐Progressive MS	5 (15.6)	8 (25)		0.73
	Relapsing Remitting MS	24 (75)	21 (65.6)		
	Secondary progressive MS	3 (9.4)	3 (9.4)		

Type of medication used	Interferon beta1	3 (9.4)	2 (6.3)		0.99
	Glatiramer acetate	3 (9.4)	4 (12.5)		
	Fingolimod	2 (6.3)	2 (6.3)		
	Dimethyl fumarate	3 (9.4)	3 (9.4)		
	Rituximab	10 (31.3)	10 (31.3)		
	Teriflunomide	2 (6.3)	2 (6.3)		
	Ocrelizumab	5 (15.4)	6 (18.5)		
	None	4 (12.5)	3 (9.4)		

Other medications	RLS medication	13 (40.6)	13 (40.6)		0.99
	Antidepressant	7 (21.9)	8 (25)		
	Both	5 (15.6)	4 (12.5)		
	None	7 (21.9)	7 (21.9)		

Age (yrs)		M ± SD (46.5 ± 9.69)	M ± SD (45.34 ± 10.39)	0.46^∗∗^	0.64

^∗^Chi‐square test.

^∗∗^Independent samples *t* test.

### 4.2. Adverse Events

Across both study groups, no harms or unintended events were observed.

### 4.3. RLS

**FIGURE 3:**
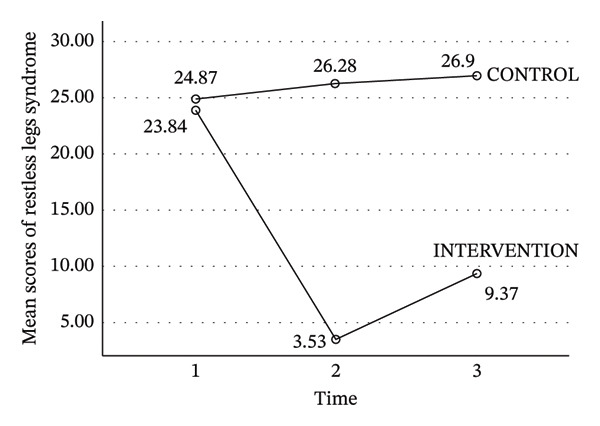
Temporal analysis of RLS in the intervention group compared with the control group in patients with MS.

There was no significant difference in the baseline RLS score between the two groups (*p* = 0.52). However, Table 2 reveals a significant difference in RLS scores between the intervention and sham groups at both the immediate postintervention and 1‐month follow‐up time points (*p* < 0.001). Specifically, the intervention group experienced a reduction in RLS scores postintervention, whereas the sham group showed an increase. Given that the trends were significant, pairwise comparisons among the time points in the intervention group were further analyzed via the Bonferroni correction. In the intervention group, there was a significant difference between the time points before the intervention and immediately after the intervention (*p* < 0.001), with the RLS score decreasing by 20.31 units after the intervention. There was also a significant difference between the time points before the intervention and 1 month after the intervention (*p* < 0.001), with the RLS score decreasing by 14.46 units 1 month after the intervention compared with before the intervention. Additionally, there was a significant difference between the time points immediately after the intervention and 1 month after the intervention (*p* < 0.001), with the RLS score increasing by 5.84 units 1 month after the intervention compared with that immediately after the intervention (Table 3). The mean RLS score in the intervention group was 22.75 units lower than that in the sham group immediately postintervention. This difference remained significant at the 1‐month follow‐up, with the intervention group scoring 17.53 units lower (Table 2, Figure 3).

**TABLE 2:** Temporal analysis of restless leg syndrome and sleep quality in patients with MS following intervention.

Variable	Before intervention	Immediately after the intervention	One month after the intervention	*F*	*p*
	Mean ± SD				
Restless Legs Syndrome					
Intervention	23.84 ± 6.64	3.53 ± 4.47	9.37 ± 6.49	97.53	< 0.001
Sham	24.87 ± 6.16	26.28 ± 6.33	26.9 ± 5.74	11.45	< 0.001
Mean difference (95% CI)		−22.75 (−25.49,‐20.01)^∗^	−17.53 (−20.59, −14.46)^∗∗^	227.35	< 0.001
Sleep Quality					
Intervention	13.12 ± 3.09	4.93 ± 3.20	6.50 ± 3.49	101.84	< 0.001
Sham	13.12 ± 3.79	13.50 ± 3.87	13.59 ± 3.50	1.196	0.306
Mean difference (95% CI)		−8.56 (−10.33, −6.78)^∗^	−7.09 (−8.84, −5.34)^∗∗^	89.37	< 0.0001

^∗^The mean difference in scores for restless leg syndrome and sleep quality between the two groups immediately after the intervention.

^∗∗^The mean difference in scores for restless leg syndrome and sleep quality between the two groups 1 month after the intervention.

**TABLE 3:** Pairwise comparisons of restless legs syndrome and sleep quality in patients with MS in the intervention group.

Variable	Comparisons	Mean difference (95% CI)	*p*
Restless Legs Syndrome	before intervention with immediately after intervention	20.31 (16.10, 24.52)	< 0.001
	before intervention with 1 month after the intervention	14.46 (10.21, 18.71)	< 0.001
	immediately after intervention with 1 month after the intervention	−5.84 (−8.54, −3.14)	< 0.001

Sleep Quality	before intervention with immediately after intervention	8.18 (6.71, 9.66)	< 0.001
	before intervention with 1 month after the intervention	6.62 (4.88, 8.36)	< 0.001
	immediately after intervention with 1 month after the intervention	1.56 (−2.94, −0.17)	0.02

### 4.4. Sleep Quality

**FIGURE 4:**
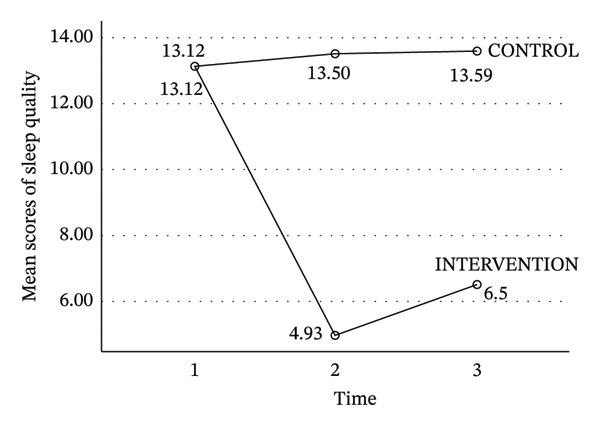
Temporal analysis of sleep quality in the intervention group compared with the control group in patients with MS.

There was no significant difference in baseline sleep quality between the two groups (*p* = 1), and a significant difference was observed at both the immediate postintervention and 1‐month follow‐up time points (*p* < 0.001). The intervention group exhibited a decrease in sleep quality scores postintervention, whereas the sham group showed no significant change. Given that the trends were significant, pairwise comparisons among the time points in the intervention group were further analyzed via the Bonferroni correction. In the intervention group, there was a significant difference between the time points before the intervention and immediately after the intervention (*p* < 0.001), with the total sleep quality score decreasing by 8.18 units after the intervention. There was also a significant difference between the time points before the intervention and 1 month after the intervention (*p* < 0.001), with the total sleep quality score decreasing by 6.62 units 1 month after the intervention compared with before the intervention. Additionally, there was a significant difference between the time points immediately after the intervention and 1 month after the intervention (*p* = 0.02), with the total sleep quality score increasing by 1.56 units 1 month after the intervention compared with immediately after the intervention (Table 3). The mean total sleep quality score in the intervention group was 8.56 units lower than that in the sham group immediately postintervention. This difference was 7.09 units at the 1‐month follow‐up (Table 2, Figure 4).

### 4.5. Sensitivity Analysis

Participants with complete symptom resolution (postintervention score = 0) were identified (Table 4). Following exclusion of these cases, the results remained statistically significant. These findings confirm the robustness of the treatment effect and suggest that the observed improvement was not driven solely by complete responders (Table 5).

**TABLE 4:** Response categories in intervention group.

Category	Definition	Approx. Percentage
Complete responders	Post = 0	47
Strong responders	Change > 15	71
Moderate responders	Change 5–15	25
Minimal responders	< 5	3

**TABLE 5:** Sensitivity analysis of temporal analysis after excluding participants with complete symptom resolution.

Variable	Before intervention	Immediately after the intervention	One month after the intervention	*F*	*p*
	Mean ± SD				
Restless Legs Syndrome					
Intervention	21.35 ± 4.74	6.64 ± 4.09	12.70 ± 5.97	33.94	< 0.001
Sham	24.87 ± 6.16	26.28 ± 6.33	26.9 ± 5.74	11.45	< 0. 001
Mean difference (95% CI)		−17.27 (−19.93, −14.61)^∗^	−12.01 (−14.95, −9.06)^∗∗^	150.81	< 0.001

^∗^The mean difference in scores for restless leg syndrome between the two groups immediately after the intervention.

^∗∗^The mean difference in scores for restless leg syndrome between the two groups one month after the intervention.

## 5. Discussion

This study demonstrated that foot reflexology significantly improved RLS and sleep quality in patients with MS. Compared with the sham group, the intervention group presented significant reductions in the mean RLS and sleep quality scores. Moreover, these improvements were sustained 1‐month postintervention. The efficacy of reflexology in alleviating RLS can be attributed to its mechanism of action on the nervous system. By stimulating sensory receptors, reflexology increases dopamine release, which is often deficient in RLS [17, 36]. A study conducted by Ghasemi et al. [17] revealed that foot reflexology reduced RLS in hemodialysis patients, whereas reflexology did not significantly decrease RLS. However, aromatherapy massage was found to be more effective than reflexology in this specific population. These discrepancies in findings may be due to factors such as patient awareness of the intervention, the number of reflexology points stimulated, and the severity of baseline RLS [17]. The lack of improvement in the control group is consistent with the findings of Ghasemi et al., in which massage without stimulation of reflexology points did not significantly affect RLS severity. This suggests that non‐specific foot massage alone is unlikely to produce a therapeutic effect on RLS symptoms. Restless legs syndrome is characterized by a fluctuating clinical course, and symptom scores may vary over time even in the absence of active intervention [37, 38]. Therefore, the slight increase observed in the sham group in the present study should be interpreted within the context of this natural variability rather than as an effect of the intervention itself.

In the present study, a substantial improvement of 20 points in the RLS severity score was observed following the intervention, indicating a marked clinical benefit. These findings of the present study align with those of Ganbari et al. [22], who reported significant improvements in RLS and sleep quality immediately postreflexology compared with a Swedish massage group. However, changes in RLS and sleep quality were not significant before and 1 month after the intervention in any of the groups, whereas in the present study, RLS scores and sleep quality remained lower in the intervention group 1‐month postintervention. This difference may be due to the daily reflexology sessions in the current study. This research demonstrated that both reflexology and Swedish massage can be effective treatments for improving RLS and sleep quality in hemodialysis patients, although reflexology is more effective. Specifically, Swedish massage led to a 5.4‐point reduction in RLS scores, whereas reflexology resulted in a markedly greater 13‐point reduction [22].

The present study demonstrated that foot reflexology resulted in an approximately 20‐point reduction in RLS scores. This magnitude of improvement is consistent with previous research. For instance, earlier studies have reported reductions of approximately 14.5 points following reflexology interventions [39], while another study evaluating foot massage with lavender aromatherapy reported a reduction of around 18 points in RLS scores [40]. Taken together, these findings suggest that clinically meaningful reductions in RLS severity—generally ranging from about 14 to 20 points—can be achieved through massage‐based interventions, including reflexology. The greater reduction observed in the present study may be related to differences in intervention characteristics, such as the number of sessions, consistency of application, and specific protocol used, which could influence the overall effectiveness of the treatment.

Reflexology increases blood flow to the affected area and promotes relaxation, which can improve sleep quality [24, 25, 41, 42]. Sajjadi et al. [12] conducted a study investigating the effects of foot reflexology on fatigue, sleep quality, and anxiety in MS patients [12]. This study, which is consistent with the present research, showed that reflexology is a noninvasive nursing intervention that can improve sleep quality in individuals with MS. Furthermore, no adverse effects were reported, suggesting that reflexology is a safe and well‐tolerated intervention. In contrast, Emamverdi et al. [21] reported that both reflexology and acupressure improved sleep quality in hemodialysis patients, although there was no significant difference between the two interventions. This may be due to the focus on specific acupoints in that study [21]. In a study by Samereh Fekri et al. [43], foot reflexology improved the sleep quality of postkidney transplant patients. In this study, the improvement in sleep quality was greater 1 week after the intervention than on the first day [43]. However, in the present study, the improvement in sleep quality decreased 1 month after the end of the intervention. Therefore, reflexology has a temporary effect, and its effectiveness decreases if the massage is not continued. In another study, Toygar et al. [44] reported that reflexology had a greater impact on anxiety than on sleep quality in caregivers of cancer patients. Similarly, the placebo effect was found to reduce anxiety in participants but was not as effective as reflexology [44]. This difference in results compared with the present study can be attributed to the short‐term intervention, which lasted only 3 days. This study also differed from the present study in terms of the study population and measurement tools.

## 6. Limitations

Owing to the massage being performed by only one person (the researcher), there was a possibility of a decrease in the quality of the massage for the patients, and efforts were made to reduce the number of patients per day to prevent this issue. Findings cannot be generalized to male MS patients. In this study, it was not possible to examine and compare the effects of reflexology massage between the two genders, and it is recommended that reflexology be studied in both genders in future research. Another limitation of this study was the sampling from a single center, as this center was the only MS association.

## 7. Conclusion

The findings of this study demonstrate the effectiveness of foot reflexology in improving RLS and sleep quality in female patients with MS. By teaching this simple and low‐cost intervention to patients and emphasizing that it does not require any special equipment and can be performed in any situation, we can improve RLS and sleep quality in patients. Additionally, owing to the lack of side effects and the beneficial effects of reflexology, patients are more willing to accept and implement it. Nurses are encouraged to learn and use foot reflexology as a noninvasive and drug‐free approach that can be easily and inexpensively performed.

## Author Contributions

Fatemeh Harandi and Batool Tirgari contributed to conceiving and designing the research. The data were collected, analyzed, and interpreted by Fatemeh Harand, Batool Tirgari, and Yunes Jahani. Batool Tirgari, Fatemeh Harandi, and Haleh Tajadini contributed equally to writing and revising the manuscript and approved the final manuscript.

## Funding

The authors received no financial support for the research, authorship, and/or publication of this article.

## Disclosure

This article was based on a master’s thesis approved by the Ethics Committee of Kerman University of Medical Sciences (Ethics Code: IR.KMU.REC.1403.143) and registered with the Iranian Registry of Clinical Trials (IRCT20151107024919N15; https://irct.behdasht.gov.ir/trial/71552) on 2023–09‐01.

## Ethics Statement

The present study was conducted after ethical approval (code: IR.KMU.REC.1403.143) from the Ethics Committee of Kerman University of Medical Sciences and a clinical trial code (IRCT20151107024919N15) from the Iranian Clinical Trial Registration Center were obtained. The principal investigator explained the study objectives and methodology to the MS patients. Written informed consent was subsequently obtained from the patients. To ensure that the participants’ information was confidential, special codes were allocated to each questionnaire, and the data were analyzed via these codes. The participants were informed that their participation was voluntary and that they could withdraw from the study at any time.

The body oil was purchased by the researcher and provided to the patients for use at home. To uphold ethical principles and address the interests of sham group participants, reflexology was provided to those who desired it at the end of the study. Additionally, the study results were reported to the relevant authorities and participants.

## Consent

Please see the Ethics Statement.

## Conflicts of Interest

The authors declare no conflicts of interest.

## Data Availability

The datasets used and/or analyzed during the current study are available, and the corresponding author can deposit the data.
